# Going blindly into the women’s world: a reflective lifeworld research study of fathers’ expectations of and experiences with municipal postnatal healthcare services

**DOI:** 10.1080/17482631.2021.1918887

**Published:** 2021-04-26

**Authors:** Bente Kristin Høgmo, Terese Bondas, Marit Alstveit

**Affiliations:** Department of Public Health, Faculty of Health Sciences, University of Stavanger, Stavanger, Norway

**Keywords:** Child and family healthcare services, expectations, experiences, fathers, reflective lifeworld research, postnatal health care, public health nurse

## Abstract

**Purpose**: The aim of this study is to describe new fathers’ expectations of and experiences with municipal postnatal healthcare services.

**Methods**: A phenomenological reflective lifeworld research (RLR) approach has been used. Ten fathers were interviewed about their expectations of and experiences with municipal postnatal healthcare services, and the data were analysed to elucidate a meaning structure for the phenomenon.

**Results**: The essential meaning of the phenomenon of fathers’ expectations of and experiences with municipal postnatal health care described as *going blindly into the women’s world*. The essential meaning is further explicated through its four constituents: not knowing what to ask for, feeling excluded, seeking safety for the family and longing for care.

**Conclusions**: Entering the postnatal period with sparse knowledge about the child and family healthcare services available is difficult for the fathers who do not know what to ask for and what to expect. The fathers’ feel excluded by the public health nurse, and the postnatal health care is seen as a mother–baby–public health nurse triad. The feeling of exclusion and inequality might be avoided if public health nurses focused both on mothers’ and fathers’ individual follow-up needs in the postnatal period and on seeing the newborn baby and the parents as a family unit.

## Introduction

Most high-income countries offer postnatal healthcare services for the families, both at the hospital of birth and in the municipalities where the families live. Early discharge from hospital for mother and child after birth (6–72 hours) is a growing trend in several countries, and this has been claimed to be a more family-centred approach in postnatal health care (Brown et al., 2009). Early discharge is found to encourage the mothers and fathers to take responsibility which contributes towards bolstering their confidence in their parental role (Nilsson et al., 2015). Many fathers can play a vital role as a parent and support person for the mother. However, the father’s role as an equal parent and caregiver may not be well enough acknowledged in cases of maternity care where the father often is named partner instead of parent (Duvander et al., 2010; Steen et al., 2012). Studies report that many fathers want to be involved in the child’s life from the very beginning (Plantin et al., 2011; Wells, 2016). Early involvement in giving care after the birth has a positive influence not only on the fathers themselves but also on their partners and on the child’s psychological, behavioural and social development and well-being (Plantin et al., 2011; Wells, 2016). The ability of the parents to share in caregiving and taking part in postnatal care might be facilitated by the availability of parental leave; in the Nordic countries, most employers offer parental leave for fathers (Sederström, 2019). Nevertheless, fewer than half of the world’s countries offer paid paternity leave on the birth of a child, and often this amounts to less than three weeks (Van der Gaag et al., 2019).

Meanwhile, studies show that expectant and new fathers often lack relevant information, guidelines and role models to support them in their transition to the fathering role and parenthood (Chin, Daiches et al., 2011; Deave & Johnson, 2008). Some fathers find their new role demanding, and new fathers’ need guidance in obtaining relevant information to prepare for the early postnatal period (Åsenhed et al., 2014; Pålsson et al., 2017). Thus, support in establishing realistic expectations of fatherhood has been highlighted as an important task for health professionals in the meeting with new fathers. Fathers want to have a relationship of trust with the public health nurse (PHN) offering postnatal health care in the municipal; they want to feel welcome and receive answers to their questions (Fägerskiöld, 2006; Wells, 2016). A father who perceives that health professionals see him only as an assistant and practical helper instead of a parent equal to the mother will feel insecure and incapable of supporting his partner (Feenstra et al., 2018; Steen et al., 2012). In a family-centred care (FCC) perspective, the partnership between the family and the healthcare provider is paramount, and studies have corroborated that this approach has improved the quality of care and resulted in increased trust and satisfaction (Coyne et al., 2018). Nevertheless, some fathers prefer support from persons other than health professionals; fathers who can talk to male workmates and friends about infant care have less need for a trustful relationship with the PHN (Fägerskiöld, 2006). Even with increased attention on the family and the paternal involvement, Hrybanova et al. (2019) report that fathers want healthcare services to treat fathers as caregivers in the consultations with the family. Becoming a father has been described by first-time fathers as an emotional roller coaster (Åsenhed et al., 2014). Communicating and sharing experiences and expectations through blogs on the internet, expectant fathers expressed that the transition to fatherhood was complex and involved both positive expectations for the future and feelings of frustration. Although some men experience psychological distress during the perinatal period, they might question the legitimacy of their experiences and feel reluctant or struggle to express their needs for support (Darwin et al., 2017). A systematic review on new parents found that depressed fathers report an imbalance between their need for support and the support they got from their partner and significant others, and it seemed like fathers had more difficulties asking for help than mothers (Holopainen & Hakulinen, 2019). Paternal depression and anxiety during the perinatal period is not just a problem for the fathers and their families; it is also a significant public health concern (Darwin et al., 2017). The literature search for qualitative studies on parents’ expectations of and experiences with postnatal health care seem almost solely to focus on mothers, and we found scarcely any studies on municipal postnatal health care for fathers (Barimani et al., 2015; Fägerskiöld & Ek, 2003; Huusko et al., 2018). This indicates a gap in the knowledge of the phenomenon of fathers’ expectations of and experiences with municipal postnatal healthcare services.

## Aim

The aim of this study was to describe new fathers’ expectations of and experiences with municipal postnatal healthcare services.

## Method

A phenomenological reflective lifeworld research (RLR) approach was chosen because RLR aims at describing the lived world in a way that increases our understanding of human beings and human experience (Dahlberg et al., 2008). The lived experiences are related to being a part of the world and human existence, characterized by an unreflected and taken-for-granted way of living and experiencing the world (Dahlberg et al., 2008). To let the phenomenon “show itself”, a sensitive openness focusing on a deliberate exclusion of assumptions and expectations is necessary (Dahlberg et al., 2008). In RLR, the researcher’s openness in the search for meaning is made visible through an attitude called “bridling” (Dahlberg & Dahlberg, 2019; Dahlberg et al., 2008). According to Dahlberg et al. (2008) bridling means having an open and alert attitude throughout the research process in order to slow down the process of understanding and going beyond our natural attitude. The researchers performing this study have clinical experience as PHNs in CFHC, and they are all mothers. Having this insider perspective, it was especially important to recognize, reflect upon and bridle our pre-understanding. By using the methodological principles referred to in RLR as openness, flexibility and bridling in the search for the phenomenon of new fathers’ expectations of and experiences with the postnatal health care, the phenomenon is explored in a scientific way focusing on not making definite what is still indefinite (Dahlberg et al., 2008).

### Setting and participants

This study is part of a larger research project that investigated new parents’ expectations of and experiences with municipal postnatal healthcare services. In Norway, municipal postnatal health care is provided for the family at CFHCs; the services are free of charge and low-threshold, following a standardized programme with regular consultations, including a home visit for newborns seven to ten days after birth. The goal of the Norwegian municipal postnatal health care is to support the parents, facilitate the parent-child attachment and mastery of the parental role. Breastfeeding and parental mental health are also important topics to be addressed by PHNs in the postnatal period (The Norwegian Directorate of Health, 2014). PHNs employed at municipal CFHCs are the primary care providers for the child and family from postnatal hospital discharge and up until the child is five years old. In addition, an early home visit by a midwife is recommended (Norwegian Directorate of Health, 2019). In Norway, more than 99% of the PHNs and midwives are women (Statistics Norway, 2019), like in Europe around 85% of all nurses are women (Boniol et al., 2019).

To gain insight and in-dept understanding of the phenomenon a purposive sampling strategy was used (Patton, 2014). Ten couples (ten mothers and fathers) participated in the study and were recruited with the help of midwives from the maternity hotel at one hospital and midwives at a CFHC in one county in the south-western region of Norway. Two couples were recruited by snowball sampling. Inclusion criteria were couples who had recently become parents, parents of different sexes, mastering Scandinavian language, mother and infant discharged from hospital within the third day after birth without the need for additional follow-up and the parents planned to utilize CFHC. One of the couples came from another Nordic country, the rest came from Norway. Six of the men were first-time fathers and four fathers were having their second child, and each father was living with the mother of the child. They were from 24 to 33 years old with a mean age of 28. The fathers’ educational level was high school (7) and university degree (3), and they were all employed and came from both urban and rural areas.

### Data collection

In order to turn to the new fathers’ lifeworld, interviews were used to explore the phenomenon of fathers’ expectations of and experiences with municipal postnatal health care. To serve the purpose of establishing an open and reflective dialogue, the interview was carried out with a focus on striking a balance between the structured and unstructured (Dahlberg et al., 2008). To direct the interviewee’s intentionality, their feelings and thoughts towards the phenomenon, the fathers were asked “can you please describe how you have experienced the first days at home with a new baby”, “what expectations do you have to the postnatal health care?” and “what experiences do you have in relation to the follow-up you and your family have received from the CFH services in the postnatal period?”. To gain a deeper understanding, follow-up questions were asked such as “can you please tell me more?” and “what has been important to you?”.

Data were collected twice, and in the first interviews, the fathers were interviewed together with the mother shortly after returning home from hospital. In the second interview, when the postnatal period was over (6 to 8 weeks after birth), the fathers were interviewed separately. In the joint interviews, the questions were directed at mother or father, and some questions were for common reflection. The two approaches complement each other in elucidating different aspects of the fathers’ expectations and experiences (Taylor & De Vocht, 2011) and conducting interviews with both the mother and father as a couple and afterwards individually provides an opportunity to both deepen and broaden the content of the data collected (Norlyk et al., 2016). When studying a phenomenon like shared experiences, joint interviews are helpful in elucidating what is often tacit knowledge (Polak & Green, 2016). In the present study the parents’ shared reflections assisted to bring new aspects into the conversation about their expectations and experiences. Another reason for conducting two interviews was to obtain expectations and experiences during the postnatal period and provide room for reflection for the fathers between the interviews. It also gave the interviewer an opportunity to follow up topics that had been discussed in the couple interview. In the joint interviews, the infant was present in the room during the interview, which to some extent affected the parents’ attention and presence. All the fathers chose their own homes as setting for the interviews.

The data collection was conducted from October 2019 to March 2020. The interviews were conducted by the first author, audio recorded and transcribed verbatim and lasted between 10 and 60 minutes (mean 35 min).

### Analysis

The interviews were transcribed verbatim and analysed according to the RLR approach described by Dahlberg et al. (2008). We strove to pursue the methodological principles of openness and flexibility, and we took a bridled attitude during the analysis process. The analysis of the data was characterized by a flexible movement between the whole and the parts, towards a new whole to describe the essential meaning structure of the phenomenon and its variations of meaning, called the constituents. The first step in the analysis process was reading and re-reading the interview transcripts to create an understanding of the whole text. This understanding served as a background on which the various parts in the individual descriptions could be understood. Moreover, through openness, the data were read with curiosity as we sought for the “otherness”, something new. Furthermore, the findings were discussed and reflected on by all authors in the search for meanings and variations of meanings, and in a dialogue including questions asked such as, “What is being said in the text?”, “How is it said?” and “What is the meaning?”. To increase the openness and reflection in the process of understanding the phenomenon, we repeatedly asked the question of whether the text could be understood in another way. Next, meaning units where identified and clustered together based on similarities and differences, forming a temporary pattern of meanings. Finally, the clustered meanings were re-read and the essential meaning of the phenomenon on an abstract level emerged. After clarifying the essence, the constituents were further described on a more concrete level and quotations from both the joint and the individual interviews were used to further illustrate the constituents. In the analyses of the data from the joint interviews, we used a non-dyadic approach and treated the data as coming from two separate individuals. The parents shared the same experience of having a baby and receiving postnatal health care in the municipality, but they may relate to those experiences differently and have different expectations regarding the encounter with the PHN and CFHC. Quotations from fathers in the joint (J) and individual (I) interviews were number coded and presented under each constituent.

### Ethical considerations

This study was designed and conducted in accordance with the principles in the Helsinki Declaration (World Medical Association Declaration of Helsinki, 2013) and was approved by the regional committee for medical and health research ethics (REC) (reg. no: 2019/7220) and by the Norwegian Centre for Research Data (NSD) (project number: 420055).)A data management plan was prepared for the project according to NSD’s template (Norwegian Centre for Research Data, 2021). All interviews were coded to guarantee participant confidentiality and the parents received both verbal and written information about the aim of the study, anonymity and the confidentiality of any data given. They were also told that they had the right to withdraw from the study at any time. The participants gave their written informed consent prior to the interviews.

## Results

The essential meaning of fathers’ expectations of and experiences with municipal postnatal health care can be described as going blindly into the women’s world. The new fathers’ enter the postnatal period with a lack of information about what postnatal care in the municipality can offer to the new family. Not knowing what to expect from the PHN and the CFHC is described as “going blindly”, and this makes it difficult to have any expectations in advance. Parenthood is seen as a joint project where both parents are important in caring for the newborn child. This stands in contrast to the manner in which the fathers perceive being met by PHN and CFHC. A feeling of being left out and excluded is predominant and adds to an image of a world *of* and *for* women which causes uncertainty about the extent to which postnatal care is a service offered to fathers as well as mothers. In the initial phase at home, fathers strive to safeguard the baby and the family, and in that context, the comfort of security is essential and serves as a necessary foundation when building a framework around the new family. An inclusive and accessible CFHC service is needed to guarantee a feeling of security. Early home visits by PHN are positive and good experiences which greatly contribute to safety. Receiving visits from competent professionals on the family’s own terms in a vulnerable transition phase is deemed a major strength. Feeling included, equal and safe is closely linked to care and while striving for the safety of their family, fathers appreciate the recognition and caring supervision of the PHN. A desire to be acknowledged and taken care of is expressed, and this underscores that fathers also need care during the postpartum period.

The essential meaning is further elaborated in the following four constituents: Not knowing what to ask for, Feeling excluded, Seeking safety for the family, and Longing for care.

### Not knowing what to ask for

The transition between hospital and home was experienced as a vulnerable phase for both the first-time and second-time fathers. The period of the first days and weeks at home with the newborn baby was described as filled with joy and gratitude, but it was also experienced as a phase entailing many questions and concerns. The fathers expressed having received little or no advance information about the CFHC offers in the postnatal period. Not knowing what the postnatal health care contained and represented for the family was experienced as going blindly and thus made it difficult for the fathers to describe their expectations. In the joint interview, one first-time father reflected upon his expectations and stated: “I don’t know exactly what to expect because I have never encountered this before. Everything is completely new” (JF5). When returning to the topic in the individual interview, the same father stated that not knowing what was going to happen when the parents came to the CFHC for the first time led to a stressful encounter with the service: “You are a bit blind when you go in there (CFHC), in relation to what is going to happen and how they do things” (IF5).

Another father expressed that he was disappointed about not having received written information about the service: “I had no expectations. I was expecting follow-up, and I got it, but—well, it was not so much in my mind. It would have been nice to get a small brochure or leaflet about the CFHC offer, in addition to the website” (JF3).

Some of the fathers’ reflections on the lack of expectations were that they needed a better knowledge base about the postnatal care and what the CFHC could offer, and wished they had this information at an earlier stage. They also described a desire to learn from other fathers’ experiences of having a baby and being able to discuss issues related to fatherhood. Sparse information about the CFHC, combined with the impression that the service’s target group was primarily the mother and the child made some of the fathers unsure as to whether or not this was an offer for fathers as well: “There is a lot that pertains to the mother and child. I’m not sure I feel that the CFHC service is an offer for me” (JF5).

Getting time off from work to accompany the mother and child to the CFHC was described as difficult by most of the fathers. Some stated that it was important that PHN planned and arranged for the father to be able to have access to the CFHC. Several fathers wanted more flexible opening hours and offers that were adapted to the fathers’ interests and needs:

I think that courses such as COS (Circle of Security), are very important to arrange so that both parents can go. It would also be nice to have a check-up dedicated to the parent who didn’t normally go to the CFHC; that would have been cool … I do not think they have it, but it would have been a nice thing really. (IF6)

The fathers said that they would have liked to get more information, knowledge and guidance related to the baby’s growth, health, and well-being. This knowledge was closely linked to the feeling of security when they were alone with the baby, feeling included by the PHN and feeling equal as a caregiver: “I regretted not getting more knowledge about children’s health … to learn the highest fever that is okay and so on … when the mother is not there. I as father would have liked to know a little more about everything related to the child’s health during the first year” (JF4).

### Feeling excluded

The fathers’ experiences with the CFHC seemed to vary depending on the contact and support from the PHN during the postnatal period. The fathers said that today’s fathers are different from the generations before them and noted that today, new fathers perceive parenting as teamwork. They want to be active and present in the child’s life from the very beginning. They believe that the father is important to both the child and the mother in the postnatal period. At the same time, the fathers said that they experienced that all information and communication with PHN and CFHC went through the mother. This was described as a feeling of being left out and excluded: “I could have been more included, if they had sent me a copy of the e-mails, or if I received a letter or … because as it is now, the mother gets everything” (IF1).

The fathers regarded parenting as teamwork and having children was described as something you do together. Not being able to take part in the consultations at the CFHC was described as though the mother had a monopoly on the child’s health and development:

It is important for the CFHC to be aware that it easily can be the mother who gets a monopoly over the child’s development and well-being in the beginning. Breastfeeding is one thing, but it is these other things like what position one should put the baby down, general training for the baby and all these other things, it would have been nice if both were involved in this. I believe many fathers, including myself, think it’s nice to be involved. But you have to be given permission to get involved … given that mandate in a way. I think that the health professionals at the CFHC certainly can think through how things are carried out based on the fact that one should include the father too. (IF6)

One of the fathers had experienced that the PHN planned the home visit when the maternity leave was over, and he had to go back to work. Not being able to be there when the PHN came on a home visit made the father feel shut out and insignificant in relation to the mother and baby: “I have not spoken with her (the PHN). The home visit was the same day I started working again … they know the dates when we are going back to work—they could have planned a little better” (IF1).

One of the second-time fathers was not aware that he could accompany mother and child to the consultations at the CFHC and regretted not being informed and invited by PHN: “No, I don’t think it was expected that I should come—I don’t think so. She (PHN) should have invited me or said something at the home visit, that the father is welcome to join. Because he is, isn’t he?” (IF8).

Several fathers had experienced that the PHN did not expect the father to attend the consultations at the CFHC. They felt that it was not a “must” that they participated, but that they could join if they wanted. This might have led to a feeling of not being significant as a caregiver and gave the impression that the follow-up was for mother and child: “If it is expected that both will join, then we will join. If it is not expected, then it ends up with only the mother going—that’s how it is, that’s the reality of the situation” (IF4).

One of the second-time fathers described how he had experienced attending a group offer at the CFHC in the postnatal period with his first child: “Based on my experiences with postnatal group meetings at CFHC from the last time, there was very little directed at father. Nothing was aimed for dad—it was a waste of two hours” (JF7). Several fathers expressed that they wanted their own arena where they could meet other fathers, share experiences and talk about topics related to infancy and early childhood that concern them. A group offer for new fathers was described as a CFHC offer they would have attended if there was one. Such an offer was also seen as an arena for building relationships and preventing loneliness during paternity leave:

If the CFHC had one day a week with longer opening hours … I don’t think they have, but it would have been a really nice thing, and maybe set up a meeting or something for fathers and children … yes, encourage it, because then maybe more fathers would dare, maybe not everyone thinks that they should, that it is only the mother. (JF9)

### Seeking safety for the family

The home visit with the PHN was described as a positive experience that helped to enhance safety the first few days after returning home from hospital. One father highlighted the importance of health personnel coming home to the family: “I think it was good, and I think it was nice to be able to ask some questions. It makes you feel safe, having such competent people come to your home … yes, and I felt it was a little more on our terms then” (JF6).

One father stated that his positive experiences with the PHN at the home visit helped to lower the threshold for contacting her in the future if needed: “I wouldn’t mind calling her if I was wondering about anything … and I don’t think it would be difficult to get help either” (IF2). The same father said that the PHN brought up things he had not thought of and that she explained why the baby did different things and how the parents could help stimulate the child’s motor and cognitive development. It gave the father a feeling of security and he was confident that the PHN would inform the parents if anything was wrong or if the baby needed extra follow-up. He also experienced that it was nice when the nurse talked about things the baby already was mastering and when she pointed out everything that was positive about the baby.

Another first-time father who for various reasons did not have access to support from his own family, expressed that an available CFHC service that clearly signalized that they were a low-threshold service was very important to him, especially during the initial phase after returning home from the hospital. This provided safety and gave the father a feeling that the first weeks at home with his new-born child had gone very well:

If something happens or we have a problem, we can drive for five minutes and we are at the CFHC and there is both a PHN and a midwife. You always have someone there if you have any questions, I think that’s good. I don’t know what it would have been like if we had lived in another municipality or far from the CFHC … I think it would have been difficult. (JF3)

### Longing for care

Feeling equal was highlighted as important in the encounter with the PHN: “She asks how I feel about the baby and how I experience things … she asks and maybe she cares too—it seems that way at least … she is nice to talk to … she listens when I talk, she seems very nice” (IF2).

In the joint interviews 1–2 weeks after birth, several parents reflected on mental health and the importance of the mother and father having a conversation with professionals about the topic and extra follow-up if needed. One of the fathers described how he reacted when the topic came up at the home visit with the PHN:

Then the postpartum depression issue came up, and the PHN asked if I had been depressed before, and I thought that was a bit surprising. Suddenly, there was something that concerned me, or she wanted to investigate whether we were in the risk group … but it is nice to feel equal in a way. It was a bit uncomfortable to be asked, but it was nice. (JF6)

Although the men described it as wonderful to be a father, some fathers reported not being prepared for their own emotional reactions related to pregnancy, childbirth, and the postnatal period. Some of the fathers in the study were concerned about paternal postnatal depression and one father expressed disappointment and a sense of injustice at not being offered help and follow-up relating to his mental health:

I just haven’t been thinking about it, but I am pretty sure that I got into a depression with the first child and that it has kept going on. She (the PHN) didn’t talk much about my mental health, but she talked with the mother about her mental health, and then I automatically thought that “OK – I actually feel the way she describes” … no one has offered me anything. It feels quite wrong. (IF8)

This second-time father described how he became aware that he was depressed and probably had been so since his first son was born three years ago while listening to the conversation between his wife and the PHN at the home visit. He had never been asked by the PHN how he had experienced the transition to fatherhood and how this had affected his mental health.

When returning to the theme in the individual interview when the postnatal period was over, none of the fathers had been asked how they felt emotionally or had been offered any kind of mapping of their mental health by the PHN, and some said that they regretted not having a dialogue with the PHN regarding their emotions and mental health:

I would have liked to fill out such a form (Edinburgh Postnatal Depression Scale - EPDS) about my mental health – or, I would have felt a little more taken care of then … yes, in a good way! Felt that someone was looking after me as father as well. If I had the opportunity to fill out a form and have a conversation, I would have thought it was perfectly fine. (IF9)

Pleasantly surprised was the description a second-time father used when talking about how he reacted when the PHN in the home visit with child number two approached him as a father to find out how he was doing. He expressed that he wished he had been asked what it was like to be a father again and pointed out that both the PHN and the midwife should have asked more about how fathers feel during the postnatal period: “They should have asked more about how the fathers are doing … I would probably say that all was well—but I know now—and I have known that from the first child, that I struggle a little with the role of fatherhood, to adapt” (JF8).

Fathers who had experienced that the PHN included them in the conversation and asked how they were doing and listened to them, felt that she cared for them too. Thus, the fathers’ who felt that they knew the PHN, expressed that the relationship was important for their feeling of safety and for mastering the parental role.

Some fathers talked about the couple relationship both in the individual and joint interviews. Especially the first-time fathers were concerned with the changes that might occur when going from being lovers to becoming a family with a baby, which requires most of the parents’ time and attention. Taking care of each other and cultivating the relationship was something they wanted to give high priority. Nevertheless, one father said, when the postnatal period was over, “It feels like it’s a state of emergency in the relationship, we hold our breathe a bit. We are mom and dad for a while, not lovers. Finding time to take care of ourselves is more restricted” (IF6).

## Discussion

The aim of the present study was to describe fathers’ expectations of and experiences with municipal postnatal health care in Norway. The essential meaning of going blindly revealed that new fathers in our study entered the postnatal period with a lack of knowledge about what kind of care and follow-up the family would receive from the PHN and the CFHC during the first weeks at home with a newborn baby. This made it difficult knowing what to ask for and what to expect as a father. Because they did not get sufficient information about what was going to happen in the consultation with the PHN at the CFHC, and having the impression that the postnatal health care was primarily an offer for mother and child, the fathers felt like they were going blindly into the women’s world. Our findings illustrate that fathers perceive parenting as a teamwork where parents are dependent on each other to give the baby best possible care. The fathers see themselves as an important caregiver and wants to be present in the child’s life from the very beginning. Fathers being involved with their children contribute to better health outcomes not only for their partners and children, but also for themselves in being more involved as fathers (Plantin et al., 2011; Sarkadi et al., 2008). The more the father engages himself during the postnatal period, the stronger his attachment will be to his baby and he will participate more during childhood (Plantin et al., 2011). The PHN and the CFHC are expected to provide care and support for the new parents, contribute to early attachment to the infant and facilitate mastery of the parenting role. Despite the fathers’ views regarding their significance for the family and the CFHC’s overall goal during the postnatal period, this study reveals that the fathers’ find that they do not have the same access to the CFHC and postnatal health care as the mother. The health service’s lack of support for the parents’ perspectives on the child as a joint project might contribute to undermining the couple relationship and the “family project”.

In a caring science perspective, caring is something natural and fundamental (Eriksson, 2002). According to Eriksson (2002), all caring is formed and arises in the relationship between a person in need of care and a caregiver. In a caring relationship, the caregiver sees the other person as a unique human being, an entity of body, soul, and spirit. The fundamental idea of caring is the alleviation of suffering and the preservation of life and health (Eriksson, 1987). With such a perspective, compassion and caring for another human being arises in the encounter and in a relationship characterized by responsibility and the desire to do good. When feeling left out by the PHN and experiencing not having the same access as the mother to the postnatal health care, the basis for establishing a caring relationship is difficult. Experiencing absence of care or neglect from the health care professionals can cause unnecessary suffering (Kasén et al., 2008). A study of new mothers’ experiences of postpartum care, showed that caring in the postpartum period was experienced as “confirmatory moments of communion” which was recognized even more strongly when experiencing the absence of care (Bondas‐Salonen, 1998).

Several fathers in our study described that they had felt the PHN did not expect them to accompany the mother and child to check-ups at the CFHC. This contributed to the fathers’ feeling insignificant and excluded in a world where care and follow-up was offered “*by* women *for* women”. This finds support in a study of first-time fathers’ experiences of support from child health nurses showing that fathers’ general perception of being supported was associated with getting necessary information, practical advice when needed, and being reassured (Hrybanova et al., 2019). Because they had not been offered this kind of support, the fathers felt that the nurses treated them unequally compared to the mothers. Despite a greater focus on promoting a more gender equal society and providing the parents with the same opportunities in maternal and child health services, studies have shown that fathers often feel left out and ignored by the health care professionals (Johansson et al., 2013; Pålsson et al., 2017; Wells, 2016). Lacking knowledge and not being able to take part in the consultations at the CFHC were described by one father in the present study as if the mother had a “monopoly” over the child’s health and development. All information and communication regarding the baby took place between the mother and the PHN, which left the fathers with a feeling of being left out. The feeling of being excluded was also related to the fathers’ experience of not having the opportunity (due to work) to take part in consultations at the CFHC, for example, when the baby’s motor and cognitive development was assessed. Not being a part of these common observations between the mother and the PHN, where the mother had the opportunity to ask questions and get advice and guidance related to stimulation and interaction with the child was perceived by the fathers as unjust. Hrybanova et al. (2019) also highlights that the child health nurses’ support should be made more available and adapted to the fathers, and that a better organization of the supporting activities (consultations, visits, and groups) is needed. The fathers’ need for adapted information is supported by Darwin et al. (2017) as a common desire among fathers. In line with our findings, there was a proclaimed desire to learn from other fathers’ experiences in addition to written and online resources. The fathers in the present study expressed that they would welcome a CFHC group for new fathers’ in which they could share experiences as fathers and have the opportunity to talk about topics that concern them in connection with the pregnancy, birth and postnatal period. Such a group offer was also seen as network-building and had the potential to prevent loneliness when fathers later went on paternity leave. Alstveit et al. (2010) found in their study of maternal support in maternity leave, that the social relationship with other mothers seemed to be the most important kind of relationship for the first-time mothers. It was proposed (Alstveit et al., 2010) that the CFHC should give all first-time mothers an opportunity to participate in peer support groups in order to strengthen their social relationships.

Safety was important for the new fathers during the first days and weeks after discharging from hospital. Especially the first-time fathers described the overall well-being of the mother and child in addition to an available health care service as significant in order to feel safe and confident at home. In our study, there were various experiences of contact and support from the PHN during the postnatal period and depending on how the fathers had experienced the relationship, an attitude emerged towards the service offer. The fathers who had experienced having little or no contact with the PHN and the CFHC were generally less satisfied, while the fathers who felt more included and familiar with the PHN said that they were more satisfied with the offer that they and the family had received in the postnatal period. However, the fathers highlighted the importance of the home visit and the first meeting with the PHN after returning home from hospital and expressed that it felt safe and reassuring having a professional coming home in a phase when everything was new and both parents felt insecure. Being given the opportunity to ask questions and being listened to as an individual person in a safe and familiar atmosphere at home gave the fathers’ a sense of security. This is supported by Persson et al. (2012), where fathers stated that participation during early parenthood, being together as a family and knowing who to ask when in doubt, were essential needs for their sense of security. In addition, being acknowledged and listened to by the health professional gave a sense of participation, which in turn gave a sense of security. The present study reveals that the fathers’ who felt involved and “seen” in the encounter with the PHN, felt that she cared for them too. Being together in a caring encounter is to be there as unique in mutuality (Holopainen & Hakulinen, 2019). Although it is the PHN’s responsibility to facilitate the encounter particularly through her attitude and demeanour, an elementary understanding of “what it is like” for the fathers from their lifeworld perspective is an important starting point for the PHN in building a caring relationship. Todres et al. (2014) describe the importance of understanding and appreciating a person’s “insiderness” and see it as one important dimension in what it takes to “humanise care”. Furthermore, as we found in our study, caring for “insiderness” needs to be given more attention. What does it mean for the PHN to understand the “insiderness” of the fathers and how to meet and act on this in caring ways? Todres et al. (2014) propose as one implication for practice that “reaching towards” another person’s “insiderness” as a practice and process is often more important than knowing the details of the person’s “insiderness” and that this care calls for lifeworld knowledge and “reflective openheartedness”.

The feeling of “going blindly” is an image portraying the interviewed fathers’ experiences of missing out on information about the offer of the CFHC, a lack of knowledge about children’s health and development, and missing communication with the PHN. The core concepts of family-centred care (FCC) include information sharing, participation, collaboration, dignity, and respect (Coyne et al., 2018). In this approach, the family is viewed as a unit and the partnership between the family and the healthcare provider is a core characteristic. Our study indicates that fathers do not experience that the PHN and CFHC focus on the family as a unit in the postnatal period. According to the national guidelines (Norwegian Directorate of Health, 2019), the dialogue between the PHN and the parents must constitute the basis for providing and receiving adequate health care, guidance and support in the postnatal period. Furthermore, as a starting point in all the contacts, the PHN is supposed to map any topics that the parents may want to address and to offer care and support in collaboration with the family based on their needs and the child’s health, development and living conditions. In contrast, our study reveals that most of the fathers experienced that they were treated like second rate parents because the focus was almost solely on the mother and child’s health and well-being. This is in line with the work of Wells (2016) who found that many Swedish fathers found that they did not receive the amount and types of support from the child health field that they wanted, and that they were not recognized as a “full parent”. On the other hand, in our study there were some fathers who had experienced that the PHN was inclusive, listened and showed genuine interest in how they were doing in the role as father. These were aspects that were deemed important in building a trustful relationship with the PHN and gave the father a feeling of being cared for in his first weeks of fatherhood. This act might be seen as the PHN reaching towards the new fathers “insiderness” by inviting herself into his lifeworld (Todres et al., 2014).

The present study indicates that while striving for safety for their family, being seen and taken care of by the PHN seems important for the fathers in the postnatal period. One of the fathers described how he struggled to adapt to fatherhood, and stressed the importance of PHNs and midwives asking both first- and second-time fathers how they are doing in the postnatal period and how they have experienced the transition to fatherhood. Chin, Hall et al. (2011) found in a study of fathers’ experiences of their transition to fatherhood that fathers should be encouraged to reflect on their relationship with their own parents and their childhood during the antenatal period, because this could influence their parenting style in a positive way. The same study highlighted the need for practitioners to discuss both positive and negative changes in the couple relationship with the new parents, because some fathers had not expected the more “negative” changes. The postnatal period represents a vulnerable phase for the individual and relational well-being and is associated with increased stressors and demands. This knowledge stresses the importance of understanding the different factors contributing to a healthier or more stressful postnatal period. The individual role satisfaction of the parents is found to be a key factor that may underlie the reduced function of relationships observed in the postnatal and child-rearing period (Cohen et al., 2019).

Most of the fathers in the present study expressed a longing for care in connection with their emotional well-being. Several of the participants in our study were concerned about postnatal depression and it was experienced as an inequality in the health care offer when the fathers were not provided EPDS-screening (Cox et al., 1987) and a conversation about their mental health at the end of the postnatal period. Preventing parental depression is important in postnatal care of the family (Holopainen & Hakulinen, 2019) and thus, the mother and the father’s well-being and mental health should be addressed during the home visit after birth and be a recurring theme in all consultations to ensure a good and safe upbringing for the child. Results from a recent study on mothers’ and fathers’ lived experiences of postpartum depression and parental stress after childbirth have concluded that parental stress and depressive symptoms have a significant impact on the parents’ everyday life and interaction with the child (Johansson et al., 2020).There is also a considerable body of evidence (Kingston et al., 2012; P. Ramchandani et al., 2005; P. G. Ramchandani et al., 2008) showing that children of depressed parents represent a high-risk group in terms of medical and psychiatric problems that can debut early and continue through adulthood. This indicates the importance of health professionals’ knowledge to identify and support both parents with these conditions in the postnatal period.

## Strengths and limitations

By adopting a reflective attitude, the authors have continuously discussed the pattern of meaning that emerged throughout the analysis process, with a focus on scrutinizing our understanding in order to remain open to see in a new way and not merely confirm what is already known (Dahlberg et al., 2008). The focus of the analysis has been meaning-oriented, and the essence of the phenomenon is described on an abstract level; according to Van Wijngaarden et al. (2017), providing an essential meaning structure is considered a core-strength of phenomenological inquiry and implies a certain generalizability of the findings. Another strength of the study is that one researcher (B.K.H) conducted and transcribed all the interviews in addition to being responsible for the analysis. Women and men who have just become parents are in a vulnerable situation. This became visible in the joint interviews conducted one to two weeks after giving birth, when the parents became tired or lost focus because the baby was frequently the dominant object of their attention. This resulted in some interviews being slightly shorter than others. Moreover, one of the individual interviews was short and lasted only for ten minutes. This was mainly because the father had not been in any contact with the CFHC or the PHN during the postnatal period and therefore was unable to share more than just a few experiences of the phenomenon. However, this might be seen as a finding in itself, and even short lifeworld interviews can contribute to nuances in descriptions. The interviews were conducted in safe surroundings in the homes of the families, also resulting in some disturbing factors in the data collection such as the baby crying, older siblings returning home from kindergarten, and telephone calls. These interruptions might have influenced the conversation and the fathers’ ability to concentrate and reflect, and thus can be seen as limitations in the study. The presence of a partner has the potential to either enhance or limit the richness of the data collected (Taylor & De Vocht, 2011). In some cases, the mother was more active than the father in the joint interviews and might have dominated the discussion. On the other hand, by conducting the joint interview first, the individual interview became a new opportunity for the fathers to bring out their own lived experiences. The fact that the interviewer was a woman interviewing men about their lived experiences during the postnatal period might be a weakness. At the same time, it may be a strength that the interviewer is a PHN with experience in talking to men about having children and becoming a father. In this study no immigrant fathers were included, and the results therefore may not include the same experiences for these fathers. The study findings reflect a Norwegian context and a homogenous group of fathers in terms of education and being employed, and this may limit the transferability as fathers’ expectations and experiences of postnatal health care might depend on the culture that one is familiar with.

## Conclusion and implications for practice

This study illustrates that fathers’ expectations of and experiences with municipal postnatal health care must be seen as going blindly into the women’s world. Little or no knowledge about the CFHC offers makes it difficult to know what to ask for and what to expect. The fathers in our study feel excluded by the PHN and CFHC, a feeling which is reinforced by an impression that the CFHCs postnatal healthcare is primarily an offer provided by women to woman and their babies, seen by the father as a mother–baby–PHN triad. In our study, safety is experienced as important in the initial phase of the postnatal period and in their pursuit to safeguard the new family, the fathers long for support and care from the PHN and CFHC. Caring for the new father by reaching out to his “insiderness” and inviting oneself into his lifeworld, the PHN might help to prevent this feeling of blindness when the father encounters the postnatal period. Our study provides important knowledge that can contribute to a more inclusive, gender-equal and family-centred postnatal care in the municipalities. Based on the interviewed fathers’ experiences, both written and oral information about the PHN role and the CFHC offer in the postnatal period should be given to both parents before the birth to enhance their feeling of security when leaving the hospital. The feeling of exclusion and inequality might be avoided by focusing on the mother’s and the father’s individual follow-up needs and by seeing the newborn baby and the parents as a family unit. The concerns about paternal mental health and emotional reactions connected to pregnancy, birth and postnatal period can be seen as a signal to the municipal postnatal health care and the CFHC that new fathers psychological wellbeing must be addressed to a greater extent. Inclusion of fathers in postnatal care might be vital for development of the family, and for future generations.

More research is needed on multicultural and vulnerable fathers’ experiences in the encounter with the PHN and CFHC in the postnatal period. Furthermore, the study raises questions about PHN’s perspectives and organizational perspectives on the CFHC regarding inclusion of fathers in postnatal care.
